# Genetic Profiling of the *Plasmodium falciparum* Population Using Antigenic Molecular Markers

**DOI:** 10.1155/2014/140867

**Published:** 2014-10-22

**Authors:** Purva Gupta, Ruchi Singh, Haris Khan, Adil Raza, Veena Yadavendu, R. M. Bhatt, Vineeta Singh

**Affiliations:** ^1^National Institute of Malaria Research (ICMR), Sector 8, Dwarka, New Delhi 110077, India; ^2^National Institute of Pathology (ICMR), Safdarjung Hospital Campus, New Delhi 110029, India; ^3^Aligarh Muslim University, Aligarh, India

## Abstract

About 50% of malaria infections in India are attributed to *Plasmodium falciparum* but relatively little is known about the genetic structure of the parasite populations. The molecular genotyping of the parasite populations by merozoite surface protein *(msp1 and msp2)* and glutamate-rich protein (*glurp*) genes identifies the existing parasite population in the regions which help in understanding the molecular mechanisms involved in the parasite's drive for survival. This study reveals the genetic profile of the parasite population in selected regions across the country with varying degree of endemicity among them. We also report the prevalence of *Pfcrt* mutations in this parasite population to evaluate the pattern of drug resistance development in them.

## 1. Introduction

Malaria parasites infect about 650 million people worldwide and* P. falciparum* alone leads to almost one million deaths per year making it the most virulent parasite causing malaria. It is pertinent to develop efficient means of controlling* P. falciparum* in areas to which malaria infections are highly endemic. Since malaria infections are endemic to India, it is necessary to characterize the parasite population and know the genotypic pattern of the circulating parasite populations [1]. The identification of multiclones in the population not only helps in assessing the parasite population dynamics but also provides insights into the existing population [2]. Previous studies have revealed high polymorphism in* msp1*,* msp2*, and* glurp* genes from different regions to which malaria is endemic [3]. In a country like India to which malaria is endemic, continuous molecular surveillance of the field isolates is required to know the pattern of existing and emerging drug resistance. This knowledge is the key to effective malaria control programs.

Genotyping the* P. falciparum* populations using antigenic genes* msp1*,* msp2*, and* glurp* has been known to describe allelic variability within parasite populations and also to distinguish recrudescence from new infections of* P. falciparum* disease [4]. Mostly these polymorphic markers (*msp1* and* msp2*) have been used to assess the multiplicity of infection (MOI) for detecting the number of clones per isolate. Block-2 of* msp1* gene, block-3 or the central repetitive domain of* msp2* gene, and RII repeat region of* glurp* show large allelic polymorphism [5]. In* msp1* gene three distinct allelic families K1, MAD20, and RO33 have been described whereas* msp2* gene consists of FC27 and IC3D7 families. Several genes of* P. falciparum *have shown extensive genetic polymorphism in genetic analysis and therefore have been extensively used as markers to study the genetic diversity and MOI [6, 7].

Chloroquine (CQ) is the most widely used antimalarial drug but* P. falciparum* has established mechanisms to evade the drug pressure. It is not very clear how the CQ resistance (CQR) is implemented but point mutations in* P. falciparum* CQR transporter protein (Pfcrt), encoded by the* pfcrt* gene, have been shown to make the parasite less sensitive to CQ [8].

The purpose of the present study was to genotype the parasite population in* P. falciparum* infections in India using antigenic polymorphic markers* msp1*,* msp2*, and* glurp *and drug resistance gene, namely,* Pfcrt*, so as to assess the parasite population dynamics in the country.

## 2. Materials and Methods

### 2.1. Sample Collection

**Figure 1:**
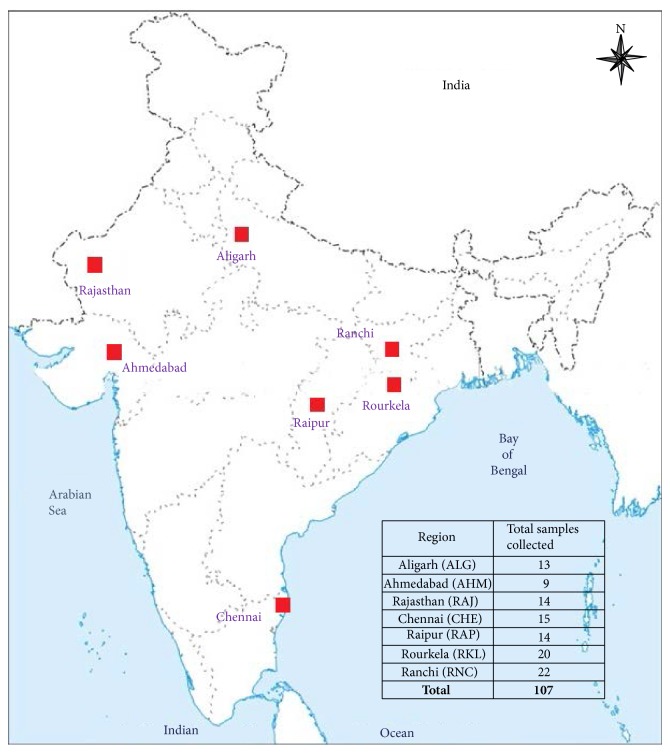
Map of India showing different regions from where malaria symptomatic samples were collected. The table specifies the number of samples collected from each region.

One hundred and seven symptomatic malaria blood samples were collected by finger prick method from different regions of India (Figure 1); the details are given in Table 1. Only after the ethical clearance from the institute the collection of* P. falciparum* infected blood samples was done. The collection of samples was carried out during the years 2009–2012.

### 2.2. *P. falciparum* Detection and Diagnosis

Microscopy and rapid diagnostic tests (RDT) were used for the first line of* P. falciparum* malaria diagnosis and bloodspots of infected samples were made on Whatman (number 3) filter paper for further molecular studies. The microscopically confirmed* P. falciparum* samples were then subjected to genotyping by different polymorphic markers.

### 2.3. DNA Isolation and PCR Analysis

Genomic DNA of* P. falciparum* positive samples was isolated by QIAamp DNA Blood Mini Kit (Qiagen Inc.) according to manufacturer's protocol. Mixed infections of* P. vivax* and* P. falciparum* were confirmed by nested PCR assay of 18srRNA primers [9].

### 2.4. Molecular Genotyping

The single* P. falciparum* infections after the species specific PCR were only considered for further molecular analysis. The repetitive polymorphic regions in different allelic families of* msp1* (block 2),* msp2* (block 3), and region II of* glurp* genes were amplified by PCR. These alleles have conserved regions flanked by repeat sequences of variable regions [5]. The primary PCR amplification of* msp1*,* msp2*, and* glurp* genes comprised an initial step of 95°C for five minutes followed by 30 cycles of 95°C for 1 minute, 58°C for 2 minutes, 72°C for 2 minutes, and a final extension of 72°C for 5 minutes. The nested PCR cycling parameters for* glurp* were the same as the primary reaction but for* msp1* and* msp2* the annealing temperature was 61°C in the nested PCR. The PCR products for* msp1*,* msp2*, and* glurp* were separated on 1.5% ethidium bromide stained agarose gels for visualization under UV illumination.

The point mutations in the* Pfcrt* gene were typed to study the polymorphisms in the drug resistance gene. In* Pfcrt* gene of* P. falciparum *the primers amplifieda fragment of 450 bp which carries SNPs from 72–76 and 97 positions. Both primary and nested PCR amplifications consisted of an initial denaturation of 94°C for five minutes with 35 cycles of 94°C for 30 s, 55°C for 30 s, 72°C for 1.5 minutes, and a final extension of 72°C for 7 minutes. Restriction fragment length polymorphism (RFLP) was carried out for the amplified region of* pfcrt* gene by enzyme* Apo I* (New England BioLabs Inc.) for the detection of SNPs as described previously with slight modifications [1, 10].

## 3. Results

A total of 87 samples were microscopically confirmed out of which 80 were found to be positive for* P. falciparum *monoinfections by PCR assay. Out of these 80* P. falciparum *monoinfections, 67 samples were successfully analyzed by PCR for* msp1*, 71 samples for* msp2*, and 51 samples for* glurp* loci in the isolates.

**Figure 2:**
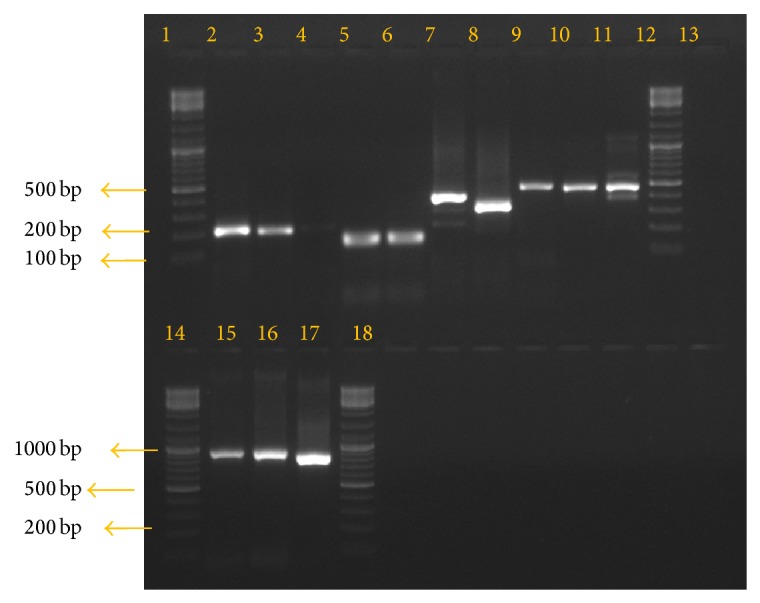
Gel picture showing the amplification of* P. falciparum msp1*,* msp2*, and* glurp* genes. Lane 2 shows* msp1* allelic family K1, lanes 3 and 4 show allelic family MAD20, lanes 5 and 6 show allelic family RO33, lanes 7 and 8 show* msp2* allelic family FC27, lanes 9, 10, and 11 show family IC3D7, lanes 15, 16, and 17 show* glurp* gene amplification, lane 13 is the negative control, and lanes 1, 12, 14, and 18 show 100 bp DNA ladder.

**Figure 3:**
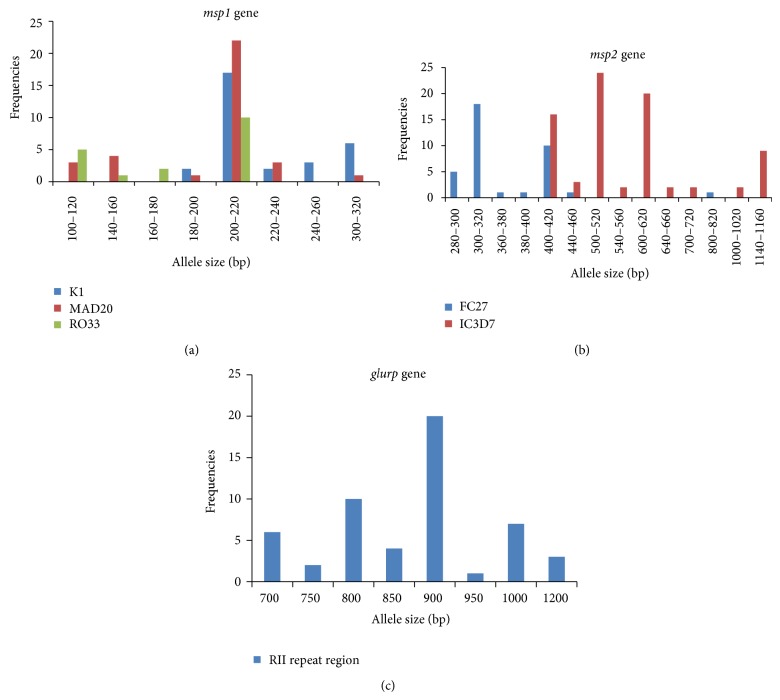
Genetic diversity of* P. falciparum* by* msp1*,* msp2*, and* glurp* genes. PCR amplification was represented by groups where PCR product size differed by 20 bp. (a) Genetic diversity by* msp1* gene. Blue bars denote the K1 allelic family, size ranging from 180 to 300 bp; red bars denote the MAD20 allelic family, size ranging from 100 to 220 bp; and green bars denote the RO33 allelic family, size ranging from 100 to 200 bp. (b) Genetic diversity by* msp2* gene. Blue bars denote the FC27 allelic family, size ranging from 280 to 800 bp and the red bars denote the IC3D7 allelic family, size ranging from 400 to 1150 bp. (c) Genetic diversity by* glurp* gene. Blue bars denote the RII repeat region of glurp gene, size ranging from 700 to 1200 bp.

After the PCR assay, the classification of the alleles was done according to the number and size of fragments and the allelic family (Figure 2). The* msp1* gene block-1 amplification for K1 allelic family was positive in 29 samples (36.25%) with five different allelic sizes ranging from 180 to 300 bp among which 200 bp allelic fragment was predominant. The other family MAD20 in* msp1 *was found in 32 samples (40.0%) depicting seven different allelic sizes within 100–300 bp and 200 bp was found to be the predominant allele size in this group. The RO33 family was detected in 17 samples (21.25%) having four distinct alleles with fragment sizes of 100–200 bp and its predominant allele was found to be 200 bp fragment (Figure 3(a)). The* msp2* amplification for FC27 family was positive in 37 isolates (46.25%) having seven different allele types ranging from 280 to 800 bp predominated by 300 bp fragment size. The IC3D7 allelic family was amplified in 45 isolates (56.25%) demarcated by nine different alleles spanning between 400 and 1150 bp fragments where 500 bp fragment was in majority (Figure 3(b)). For* glurp* 51 samples (63.75%) were found to be positive for RII repeat region producing eight different sizes ranging between 700 and 1200 bp, among which 900 bp allele was found to be predominant (25.0%) (Figure 3(c)). The number of genotypes for each marker is shown in Tables 2 and 3.

It was seen that 46.25% of the isolates studied were multiclonal in nature with two or more alleles present in* msp1*,* msp2*, and* glurp* genes. Thirteen multiple alleles were seen in* msp1* (16.25%), 28 were found in* msp2* (35%), and two multiple alleles were seen in* glurp* (2.5%) genes showing more genotypic variation in* msp2* than in* msp1* and* glurp*. There was only one isolate from Rourkela, positive for all the allelic families of* msp1* and* msp2*. There were 21 distinct haplotype patterns observed among the parasite population and the prominent genotype pattern was MAD20 for* msp1* and FC27 for* msp2*.

**Figure 4:**
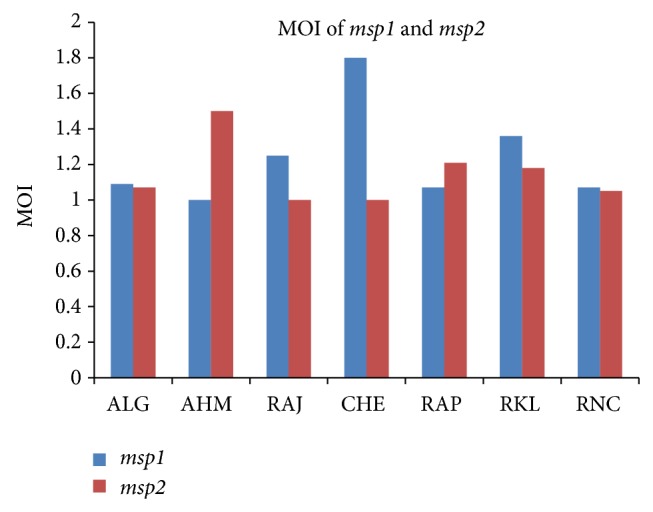
Graph showing comparison of MOI for* msp1* and* msp2* genes in different regions of India. ALG: Aligarh, AHM: Ahmedabad, RAJ: Rajasthan, CHE: Chennai, RAP: Raipur, RKL: Rourkela, and RNC: Ranchi.

MOI is calculated as the number of genotypes for a gene divided by the number of isolates with positive PCR amplification. For each region studied MOI of both* msp1* and* msp2* was calculated whose results are shown in Figure 4.

A fragment of 450 bp in* pfcrt* gene was amplified for 80* P. falciparum* isolates out of which 68 samples showed successful amplification. Restriction sites for* Apo I* enzyme are seen at codon 76 where K76T mutation was observed in 29.4% of the isolates.

## 4. Discussion

Though, for parasite genotyping,* msp1*,* msp2*, and* glurp* genes have been the recommended molecular markers for several drug efficacy studies, still the parasite population genetic profile has not been assessed systematically in a country to which malaria is endemic like India [6, 11, 12]. The field isolates in the present study have been genotyped using the polymorphic regions of these three genes in order to compare the diversity and the existing allelic frequencies.

The* msp1 *and* msp2 *showed 16 each, and* glurp* markers showed eight allelic families in the studied parasite population. These findings indicate that, for detection of MOI,* msp1* served as a better marker as MOI for* msp1* was higher in comparison to* msp2*. However it is noticeable that* glurp* did not indicate the existing genetic pattern with lower frequency of diverse alleles [13]. This study shows the complex diversity existing in the* P. falciparum* field isolates in areas of the country where malaria is endemic. The diversity seen in these isolates is due to the complexity present in the parasite population.

Previously it has been reported that a higher MOI was found in severe infections as compared to the mild type infections in studies from Uganda [14]. These findings were in tandem with studies from India, which showed a strong association between multiple genotype infections, significantly high MOI, and severity of* P. falciparum* malaria [15, 16]. Findings from Yemen show a regional variation with a higher MOI in isolates from foothills/coastland areas as compared to those from the highlands [17]. Another study from India showed a high proportion of multiclonal isolates and high MOI in regions to which* P. falciparum* is highly endemic [18]. Findings on the genetic pattern of these polymorphic genes* msp1*,* msp2*, and* glurp* of field isolates are similar to the other studies reported elsewhere [3, 4]. Studying the dynamics of multiclone infections and MOI in relation to the host immunity, disease prevalence, genetic structure, and geographical distribution would allow us to know more about the virulence of* P. falciparum*.

We were able to identify 21 distinct genetic patterns among the parasite population indicative of the fact that a considerable amount of gene flow is ongoing between the different regions with* P. falciparum* infections. It would be interesting and of importance to link the genotypic pattern of the parasites with the clinical phenotype. The recent data of* msp1*,* msp2*, and* glurp* markers for drug efficacy studies are highly important in areas where malaria is endemic for understanding the treatment criteria [19]. These highly polymorphic surface proteins are vaccine candidate genes and monitoring these genes to understand their genetic diversity and structure would help us develop vaccines with cross-protection against a range of antigenic variants [13, 20].

The studied drug resistance gene indicates that extensive mapping of this gene should be carried out in more areas in which malaria is endemic. The resistant mutants are the result of drug pressure and hence control programs should be devised in such a manner that they help in reducing the drug pressure and enhance the activity of antimalarials. This study indicates the high complexity and the diversity existing in the* P. falciparum* population in several areas of the country where malaria is endemic. The MOI was higher in* msp1* in this reported study, suggesting that the intensity of malaria transmission in these regions is high. This multiplicity of infection has definite implications not only in the drug resistant parasite but also in the outcome of the disease treatment. The diversity (MOI) in the parasite population was seen more in the rural areas like Rourkela and Ranchi than in urban areas like Ahmedabad and Aligarh. This difference in the genetic diversity could be attributed to the topographical and climatic changes in the environmental factors. This kind of study in addition to other molecular methods should be undertaken for monitoring the existing and emerging drug resistance patterns in malaria disease.

## Figures and Tables

**Table 1:** Summary of the *P. falciparum* isolates collected from various regions across India.

S. number	Region	Total samples collected	Samples positive for *P. falciparum* by microscopy and RDT	*P. falciparum* monoinfections by PCR
1	Aligarh (ALG)	13	13	13
2	Ahmedabad (AHM)	9	9	9
3	Rajasthan (RAJ)	14	4	4
4	Chennai (CHE)	15	5	5
5	Raipur (RAP)	14	14	14
6	Rourkela (RKL)	20	20	13
7	Ranchi (RNC)	22	22	22

	Total	107	87	80

**Table 2:** Region-wise genetic diversity of *P. falciparum* by *msp1*, *msp2*, and *glurp*.

S. number	Region	Number of samples (*n*)	PCR assay							
			*msp1 *				*msp2 *			*glurp *
			K1	MAD20	RO33	Total	FC27	IC3D7	Total	
1	Aligarh	13	2	7	3	12	9	5	14	10
2	Ahmedabad	9	1	2	4	7	7	5	12	8
3	Rajasthan	4	1	2	2	5	3	1	4	3
4	Chennai	5	2	4	3	9	3	1	4	3
5	Raipur	14	8	5	2	15	9	8	17	12
6	Rourkela	13	9	3	3	15	5	8	13	11
7	Ranchi	22	6	9	0	15	1	17	18	4

**Table 3:** Summary of all the isolates positive for each allelic family for *msp1*, *msp2*, and *glurp* along with the number of alleles and the range of sizes of alleles.

Loci	Number of samples positive by PCR	Number of distinct alleles	Sizes of alleles (bp)
MSP-1			
K1	29	5	180–300
MAD20	32	7	100–300
RO33	17	4	100–200
MSP-2			
FC27	37	7	280–800
IC3D7	45	9	400–1150
GLURP	51	8	700–1200
